# Impairment of Visual Function and Retinal ER Stress Activation in *Wfs1*-Deficient Mice

**DOI:** 10.1371/journal.pone.0097222

**Published:** 2014-05-13

**Authors:** Delphine Bonnet Wersinger, Nesrine Benkafadar, Jolanta Jagodzinska, Christian Hamel, Yukio Tanizawa, Guy Lenaers, Cécile Delettre

**Affiliations:** 1 INSERM U1051, Institut des Neurosciences de Montpellier, France and Université de Montpellier I et II, Montpellier, France; 2 Centre Hospitalier Universitaire, Genetics of Sensory Diseases, Montpellier, France; 3 Division of Endocrinology, Metabolism, Hematological Sciences and Therapeutics, Yamaguchi University Graduate School of Medicine, Yamaguchi, Japan; Ecole Normale Supérieure, France

## Abstract

Wolfram syndrome is an early onset genetic disease (1/180,000) featuring diabetes mellitus and optic neuropathy, associated to mutations in the *WFS1* gene. *Wfs1−/−* mouse model shows pancreatic beta cell atrophy, but its visual performance has not been investigated, prompting us to study its visual function and histopathology of the retina and optic nerve. Electroretinogram and visual evoked potentials (VEPs) were performed in *Wfs1^−/−^* and *Wfs1^+/+^* mice at 3, 6, 9 and 12 months of age. Fundi were pictured with Micron III apparatus. Retinal ganglion cell (RGC) abundance was determined from Brn3a immunolabeling of retinal sections. RGC axonal loss was quantified by electron microscopy in transversal optic nerve sections. Endoplasmic reticulum stress was assessed using immunoglobulin binding protein (BiP), protein disulfide isomerase (PDI) and inositol-requiring enzyme 1 alpha (Ire1α) markers. Electroretinograms amplitudes were slightly reduced and latencies increased with time in *Wfs1^−/−^* mice. Similarly, VEPs showed decreased N+P amplitudes and increased N-wave latency. Analysis of unfolded protein response signaling revealed an activation of endoplasmic reticulum stress in *Wfs1* ^−/−^ mutant mouse retinas. Altogether, progressive VEPs alterations with minimal neuronal cell loss suggest functional alteration of the action potential in the *Wfs1* ^−/−^ optic pathways.

## Introduction

Wolfram syndrome (WS) (OMIM 222300) is a rare multisystem disorder (1/160,000 in Europe; 1/100,000 in North America) featuring systematically early-onset type I diabetes mellitus and optic neuropathy, variably associated with neurological dysfunctions [1], [2], [3], [4]. Patients often develop in their second decade sensori-neural hearing impairment, diabetes insidipus and urinary dysfunctions. Psychiatric illness impairs live of most diagnosed patients and sometimes their relatives, as heterozygous carriers [5], [6]. WS patients have a median lifespan of 30 to 40 years, and often decease from central respiratory failure or dysphagia.

WS is an inherited condition transmitted by recessive point, frame-shift and missense mutations in *WFS1* gene, which consists in 8 exons and extends on 33.4 kb of chromosome 4 q. In 2010, 219 WS patients had been described with 172 different reported mutations in *WFS1*, mostly (83%) in exon 8 [7], [8], [9]. Patient genotype do not present any mutation hotspot, highlighting the need to sequence first exon 8 for molecular diagnosis [10]. *WFS1* translation starts in the second exon and produces a 890 aminoacid protein named Wolframin, consisting in 9 transmembrane domains, surrounded by large N- and C-terminal regions.

Eye fundus examination of WS patients reveals a bilateral optic disc pallor as a consequence of optic nerve atrophy [2], [11]. Magnetic resonance imaging discloses visual system atrophy at the central level, with reduced signals in the optic nerves, chiasm and tracts [12]. Consequent to anatomic loss, visual impairment is diagnosed by reduced visual field and pattern visual evoked potentials (P-VEP) [1], [13], [14], [15]. A progressive decrease of visual acuity associated to visual field scotomas lead to legal blindness. In human retina, Wolframin is detected in retinal ganglion cell (RGC) soma and axons, and in non-myelinated proximal optic nerve (ON) [16].

Wolframin was described as a protein of the secretory pathway, mostly located in the membrane of the endoplasmic reticulum (ER), but also in insulin secretory granules of pancreatic beta cells. Its function is assumed to regulate a ionic channel involved in calcium homeostasis [17], [18], contributing to the acidification of insulin secretory granules [19]. In agreement with the modulation of insulin release, *Wfs1*-deficient mice present an impaired glucose homeostasis, due to a progressive loss of pancreatic islet beta cells [20]. Glucose dysregulation affects cell cycle of *Wfs1*-deficient beta cells and increases their apoptosis, as a consequence of increased ER stress response [21].

As an intracellular echo of ER dysfunctions, the Unfolded Protein Response (UPR) is an adaptive cellular response that prevents cell damages and apoptotic mechanisms. Three signaling pathways, initiated by dissociation of BiP chaperone (Binding immunoglobulin Protein) at its luminal part from 3 effectors, are involved in the UPR : PERK (PKR-like ER kinase), IRE1 (inositol requiring 1) and ATF6 (Activating Transcription Factor 6) [22]. ER membrane proteins like Wolframin participate to the UPR [23], [24]. Indeed, *WFS1* gene expression was shown to be promoted by ER stress, with increased IRE1 and PERK activities [25], [26]. More importantly, Wolframin was shown to prevent ATF6α transcriptional activity in pancreatic beta cells, through the ubiquitin-proteasome pathway and to repress ATF6α-mediated activation of ER stress response promoters in the nucleus [27]. In contrast, UPR signaling is not activated in WS asymptomatic tissues, as heart, skeletal muscle and brown adipose tissues of *Wfs1-*deficient mouse [21].

Cell death associated to ER stress was identified in many central neurodegenerative conditions, including blinding diseases as hypertensive glaucomas, diabetic retinopathies, retinitis pigmentosa and age-related macular dystrophies. In mouse, Wolframin is expressed in the retina, with predominance in RGC and inner nuclear layer, and could prevent UPR signaling. Looking for a mouse model for WS optic neuropathy, we analyzed visual function of *Wfs1* knock-in mice modified in *Wfs1* translation starting site and described as a model of type 1 diabetes mellitus with normoglycemia and hypoinsulinemia [20]. In our longitudinal analysis, significant impairments of retinal function and visual signal propagation to the brain at 9 and 12 months were found by electrophysiology. All other visual parameters appeared unchanged, given eye fundi, visual acuity and contrast sensitivity. UPR signaling was activated in mutant retinas, without significant loss of RGC soma or axons, but possibly linked to the progressive visual impairment in *Wfs1*-deficient mouse.

## Materials and Methods

### Mice

All experimental procedures adhered to the ARVO Statement for the use of Animals in Ophthalmic Research and to European Union directives. All protocols were conducted under the project agreement number n°CEEA-LR-12123 given by the Languedoc Roussillon Comity of Ethics in Animal Experimentation (CEEA-LR). *Wfs1*-deficient mice bred on C57/Bl6J genetic background carried a neomycin cassette disrupting the translation starting site in the second exon of *Wfs1* [20]. Genotyping was conducted as previously described [20]. Animals were kept in a 12 h light-dark cycle with food and water available *ad libitum*.

### Electrophysiology

Electrophysiological examinations were performed under dim-red light at morning hours on dark-adapted animals. Pupillary dilatation was induced by 0.5% tropicamide instillation (Mydriaticum, Théa, France) in both eyes. Anesthesia was obtained by intraperitoneal injection of ketamine (45 mg/kg body weight; Merial, France) and xylazine (17 mg/kg bodyweight; Bayer Healthcare, Germany). Electroretinogram (ERGs) recordings performed with cotton wick electrodes as previously described [28] were acquired through Visiosystem device (SIEM Bio-Medicale, France). For ERGs, mice were subjected to sequences of 7 repeated flashes (5 msec- long flashes at 0.3 Hz frequency) and flash intensity (0.159, 0.3, 0.5, 1.59, 5, 15.9, 50 and 159 cd.s.m^–2^) was increased between phases to test rod photoreceptor function (scotopic testing) prior to both populations of photoreceptors (mesopic testing). Maximal intensity was used to illuminate animals when measuring visual electric potentials (VEP; 5 msec-long flashes at 1 Hz frequency; 60 flashes). Electroretinograms were performed on 7 to 14 animals of both genotypes at 3, 6, 9 and 12 months; VEP were conducted on 9 to 20 mice, with 3 to 5 traces collected per animal.

### Visual Acuity and Contrast Sensitivity

Vision was assessed by a behavioral test referred as the visual optomotor task. Rotating black and white stimuli were projected at definite frequencies and contrasts around the mouse with the OptoMotry system (Cerebral Mechanics, Alberta, Canada). Head tracking reflex was detected until the stimuli was not perceptible by the animal, thus allowing to measure frequency and contrast thresholds as a characterization of a mouse spatial vision. Mice were adapted to a central platform for 2 minutes in a grey environment. For frequency threshold test, 100% contrast sinusoidal stimuli were rotating around the animal with increasing frequencies (from 0.042 to 0.4 c/d) until the reflex was lost. For contrast sensitivity test, mice were exposed to 0.1 c/d rotating stimuli with decreasing contrast, until the contrast threshold was reached. 10 to 15 mice were tested at 3, 6, 9 and 12 months for each genotype.

### Eye Fundus

Eye fundi were captured using the Micron III retinal imaging system (Phoenix Research Labs, Pleasanton, CA). Pupillary dilatation and anesthesia were induced as described above.

### Immunochemistry

Eyes and optic nerves (n = 3 to 4) were enucleated and fixed in cold 4% paraformaldehyde overnight. After tissue cryoprotection and inclusion, 12 µm sagittal sections were collected in the mid-retina, at the optic nerve head. Frozen sections were stained with haematoxylin and eosin or permeabilized by 0.1% Triton X-100 for immunohistochemistry. Non-specific binding sites were blocked by 10% normal donkey serum. Goat polyclonal anti-Brn3a antibodies (Santa Cruz Biotechnology, Heidelberg, Germany) were used to label RGC nuclei. Total retinal nuclei and Brn3a positive RGC of 6 labeled retinal sections were pictured on Nanozoomer slide scanner coupled to fluorescence filters (Hamamatsu Photonics France, Massy France). Primary antibodies used were: WFS1 (Euromedex, Souffelweyersheim, France), Phospho-Ire1α (Novus Biologicals, Cambridge, UK), NF200 (Sigma, Saint Louis, USA) and LC3 (Sigma, Saint Louis, USA). Microscope slides were visualized using a Zeiss, LSM 510 Meta confocal microscope. The intensity of LC3 immunolabeling in RGC was quantified from confocal images using custom-made program written in MATLAB (The Mathworks). The LC3 labeled voxels (red channel) localized in RGC were isolated. The range of fluorescence intensity per pixel in the RGC layer was from 0–255∶0 =  black, 255 =  saturated. Mean intensities were calculated in wild type (+/+) and mutant (−/−) mouse.

### Transmission Electron Microscopy (TEM)

Myelinated optic nerves of 12 month old animals (n = 3–4) were fixed overnight in 3.5% glutaraldehyde (0.1 M, pH 7.4 in Sorensen’s buffer), then washed in Sorensen’s buffer and post-fixed in a 1% osmic acid for 2 h in the dark at room temperature. After two rinses, nerves were dehydrated in a graded series of ethanol solutions (30–100%) and embedded in EmBed 812 using an Automated Microwave Tissue Processor for Electronic Microscopy, Leica EM AMW. Transversal thin sections (80 nm; Leica-Reichert Ultracut E) were collected at different levels of each block, counterstained with uranyl acetate and observed using a Hitachi 7100 TEM (Centre de Ressources en Imagerie Cellulaire de Montpellier, France). Axon total numbers were counted in 10 images of 111 µm^2^, pictured randomly in the transversal section of the optic nerve.

### Q-PCR Assay

Eyes of *Wfs1* ^+/+^ and *Wfs1* ^−/−^ mice were enucleated and dissected rapidly in order to flash freeze retinal tissues. Total RNAs of one frozen retina per animal at 3, 6, 9 and 12 month old were purified using RNeasy mini kit (Qiagen, Courtaboeuf, France) according to manufacturer’s protocol. Equal amounts of RNA were reverse transcribed using the Superscript III First Strand Synthesis System (Life technologies, Saint-Aubin, France). Quantitative PCRs (qPCR) were performed on the LightCycler 480 thermocycler with SYBR Green detection (Roche). Relative RNA quantities of *Nrn1* and *Thy1* were normalized to *L27* gene values. Primers used for amplification were (from 5′ to 3′): *L27*-F : ACGCAAAGCCGTCATCGTGAAG, *L27*-R : CTTGGCGATCTTCTTCTTGCC; *Nrn1*-F: TATTTCACTGATCCTCGCGG, *Nrn1*-R : GAAATCCTCCCAGTATGTGC; *Thy1*-F: CTCCTGCTCTCAGTCTTGC, *Thy1*-R : CCTGAGAGCACGTGCTTCC, *CHOP*-F: TATCTCATCCCCAGGAAACG, *CHOP*-R: CTGCTCCTTCTCCTTCATGC, *Grp78/Bip-*F: TTCAGCCAATTATCAGCAAACTCT, *Grp78/Bip*-R: TTTTCTGATGTATCCTCTTCACCAGT, spliced *Xbp1*-F (sXbp1F): GAGTCCGCAGCAGGTG, spliced *Xbp1*-R (sXbp1R): GTGTCAGAGTCCATGGGA.

### Western-Blotting

For ER stress studies, one frozen retina was used per animal (n = 6 per genotype) at 12 month old. Retinas were lysed in 1% Triton X-100, 250 mM NaCl, 5 mM EDTA, 50 mM Tris HCl pH7.5, completed with protease and phosphatase inhibitors (Roche Applied Science, Meylan, France). Proteins of NIH3T3 mouse embryonic fibroblasts, treated by 500 nM thapsigargin served as ER stress controls [29]. Protein lysates were separated by migration on Ready Tris-HCl 10% polyacrylamide gels in Tris-glycine-SDS buffer (BioRad, Marnes-la-Coquette, France). After protein transfer to PVDF membranes, and saturation of non-specific binding sites with dry milk, membranes were incubated with specific primary antibodies. Horse raddish peroxidase-conjugated secondary antibodies were detected with enhanced chemiluminescence kit (ECL prime, GE Healthcare Europe Gmbh, Velizy-Villacoublay, France) using the Molecular Imager Gel Doc XR System (Bio-Rad) and analysed using Image J software. Primary antibodies used in our experiments were anti-beta actin (Sigma, Lyon, France), anti-GRP78/BiP (Abcam, Paris, France), anti-PDI (Cell Signaling Technologies, Danvers, MA), anti-Ire1α (CST), PERK (Cell Signaling, Danvers, USA), Phospho_PERK (Santa Cruz Biotechnology, Heidelberg, Germany), ATF6 (ABCAM, Cambridge, UK) and LC3 (Sigma, Saint Louis, USA).

### Statistical Analysis

Wild-type and KO data were analyzed for statistical significance by a Student non-parametric mean comparison (*, p<0.05).

## Results

**Figure 1:**
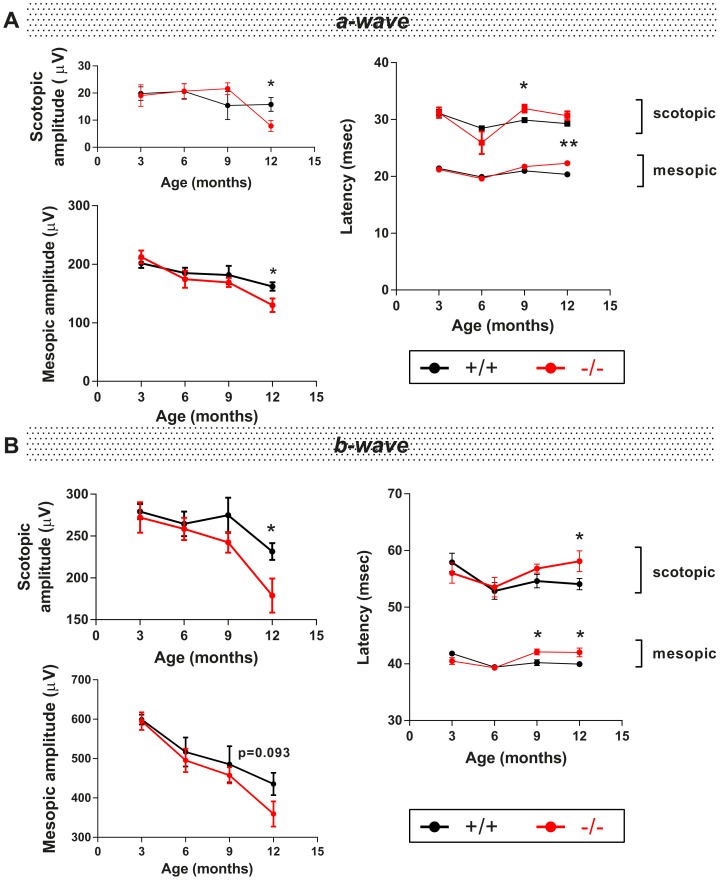
Photoreceptor and inner retinal functions in *Wfs1* ^−/−^ mice. Photoreceptor and inner retina electroretinogram data are presented in panels A and B, from ERG a- and b-wave respectively, on *Wfs1* ^+/+^ and ^−/−^ mice. Both waves are represented by mean values of amplitudes and latencies at the ages of 3 (n = 10 *Wfs1* ^+/+^, n = 8 *Wfs1* ^−/−^), 6 (n = 8 *Wfs1* ^+/+^, n = 7 *Wfs1* ^−/−^), 9 (n = 12 *Wfs1* ^+/+^, n = 14 *Wfs1* ^−/−^) and 12 months (n = 10 *Wfs1* ^+/+^, n = 10 *Wfs1* ^−/−^) in scotopic and mesopic conditions. Black and red traces correspond to *Wfs1^+/+^* and *Wfs1^−/−^* animals respectively. Statistical significance is indicated when p<0.05 (*) and 0.01 (**).

Rod photoreceptors (PR) are the major sensor of light in mouse as they constitute 97.2% of photoreceptors [30]. To characterize rod PR function in *Wfs1* mutant mice, flash ERG were recorded in 3, 6, 9 and 12 month old mice after dark adaptation, in mesopic conditions. A-wave, depicted in Figure 1A, was significantly reduced in *Wfs1^−/−^* mice (Figure 1A), with a 19.7% loss of mixed PR response in mesopic conditions. Latencies of mutant mice were also increased by 9.63% in mesopic conditions at 12 months. Finally, non-significant differences in scotopic traces were found in 9 and 12 month old animals, probably resulting from small A-wave amplitude.

B-wave (Figure 1B) amplitudes of scotopic adapted *Wfs1^−/−^* mice were significantly reduced at 12 months, with a peak delayed by 7.5%. Scotopic inner retina response was progressively altered in mutant mice with 11.8% reduction at 9 months and a significant 22.7% reduction at 12 months. Similarly, mesopic b-wave amplitude appears progressively affected in *Wfs1^−/−^* mice with a faint decrease at 6 and 9 months and a 17.5% impairment at 12 months (p = 0.093). At this age, mesopic inner retina was found less activated by 1.59, 15.9, 50 and 159 cd.s.m^–2^ flash stimulations in *Wfs1^−/−^* mice, with p-values very close or below significance threshold (p = 0.048, 0.029, 0.064 and 0.093 respectively, data not shown). Mesopic b-wave were also significantly delayed in mutant group at 9 and 12 months (5.2% delay for both). Retinal functions of *Wfs1* ^−/−^ animals are thus dysfunctional at 12 months, particularly for ERG b-wave response of the inner retina.

**Figure 2:**
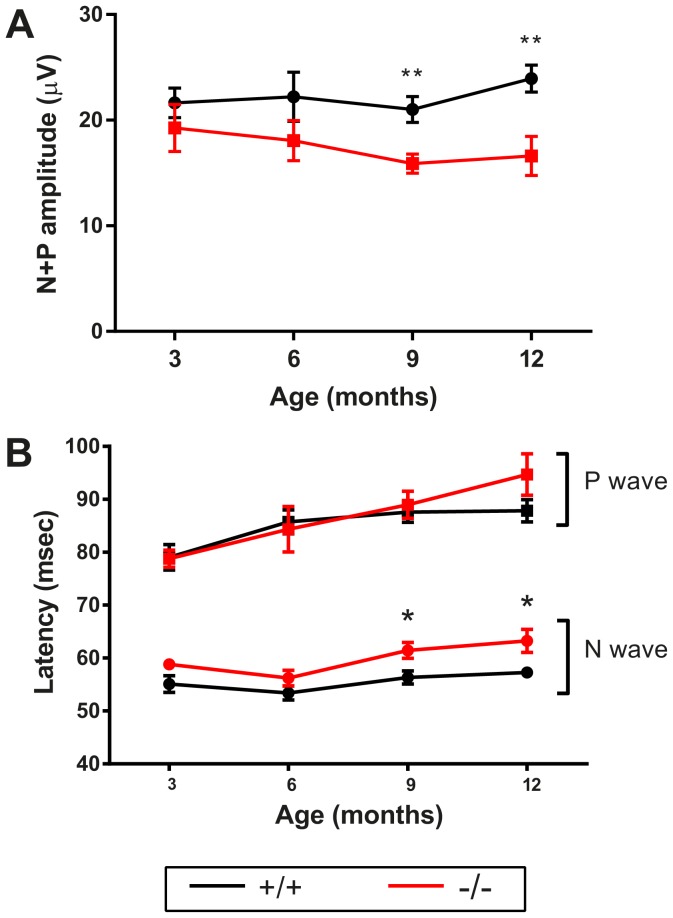
Light-induced electric conduction from retina to visual cortex. Visual evoked potentials measured from 3 to 12 months are depicted as average +/− SEM of N+P amplitudes (A) and N- and P-wave latencies (B). Black circles and red squares correspond respectively to *Wfs1* ^+/+^ and *Wfs1* ^−/−^ animals. Statistical significance is indicated when p<0.05 (*) and 0.01 (**). For 3 and 6 months n = 9 for *Wfs1* ^+/+^ and *Wfs1* ^−/−^, for 9 months n = 13 *Wfs1* ^+/+^ and n = 20 *Wfs1* ^−/−^, for 12 months n = 14 *Wfs1* ^+/+^ and n = 9 *Wfs1* ^−/−^.

To assess electric conduction along visual system, from retinas to visual cortices, flash visual evoked potentials were measured immediately after ERG in mesopic conditions. Young *Wfs1^−/−^* mice, at 3 and 6 months, presented no modification of their VEP traces in terms of amplitudes and latencies. At 9 and 12 months, VEP amplitudes in *Wfs1^−/−^* mice were significantly reduced by 24 and 30% respectively (Figure 2A). Moreover, a significant 5 percent increase of n-wave latency (Figure 2B) was observed at 9 and 12 months, suggesting a slower conduction along the visual pathways.

**Figure 3:**
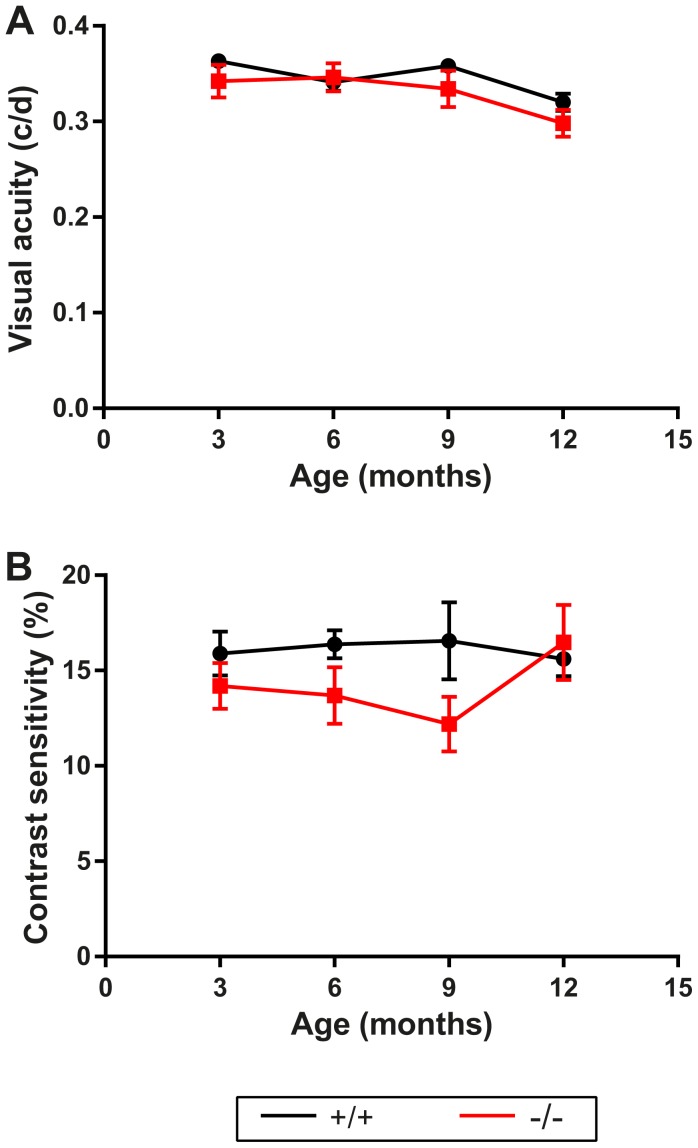
Behavioral analysis of subcortical vision. Visual acuity (A) and contrast sensitivity (B) measured by the visual optomotor task are shown as averages +/− SEM at 3, 6, 9 and 12 months. Black and red traces correspond respectively to *Wfs1* ^+/+^ and *Wfs1* ^−/−^ animals. Significance (*) is indicated when p<0.05. For visual acuity: for 3 months animals n = 11 *Wfs1* ^+/+^ and n = 13 *Wfs1* ^−/−^, for 6 months n = 12 *Wfs1* ^+/+^ and n = 14 *Wfs1* ^−/−^, for 9 months n = 10 *Wfs1* ^+/+^ and n = 7 *Wfs1* ^−/−^, for 12 months n = 15 *Wfs1* ^+/+^ and n = 10 *Wfs1* ^−/−^. For contrast sensitivity: for 3 months animals n = 9 *Wfs1* ^+/+^ and n = 13 *Wfs1* ^−/−^, for 6 months n = 9 *Wfs1* ^+/+^ and n = 9 *Wfs1* ^−/−^, for 9 months n = 11 *Wfs1* ^+/+^ and n = 7 *Wfs1^−/−^*, for 12 months n = 15 *Wfs1* ^+/+^ and n = 9 *Wfs1* ^−/−^.

To determine if visual acuity was affected, *Wfs1^−/−^* mouse vision was also evaluated by a behavioral test measuring the optokinetic tracking response. 5.8 to 6.9% decreases of frequency thresholds were found in *Wfs1^−/−^* mice, as compared with controls at 9 and 12 months, but without statistical significance (Figure 3A). High variability was found in optokinetic tracking response when testing contrast sensitivity, but *Wfs1^−/−^* disrupted mice tended to see lower contrasts, better than did controls (Figure 3B). A central nervous system (CNS) damage in *Wfs1^−/−^* mice could in turn be ineffective in triggering proper visually-evoked behavioral responses. In addition, it should be noted also that *Wfs1^−/−^* mice nervosity could lead to a biased visual acuity assessment, since mutant mice elicited large tracking movements, whereas wild type animals displayed more discrete responses.

**Figure 4:**
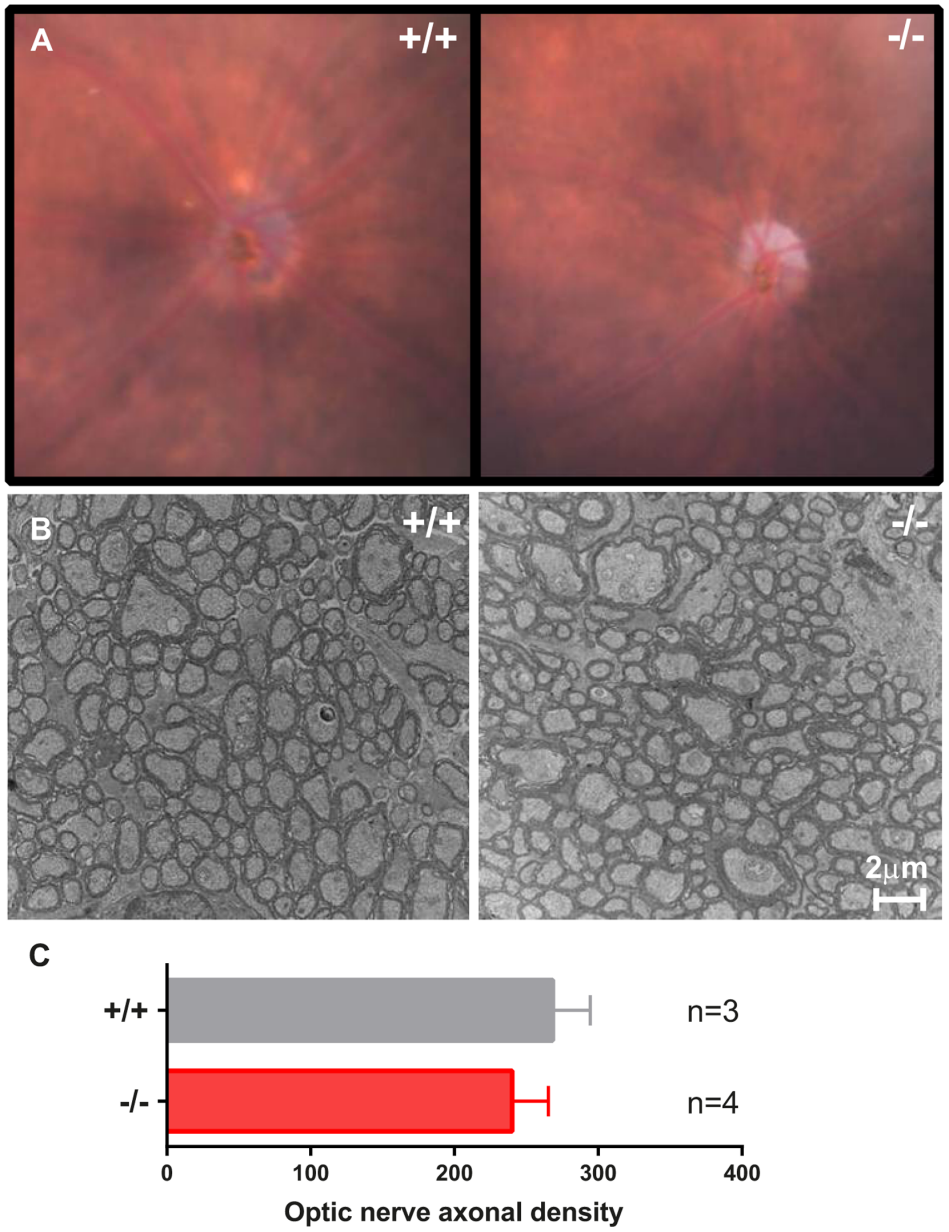
RGC axons at the optic disc and in the optic nerves. Representative eye fundus of 12 month old *Wfs1^+/+^* and *Wfs1* ^−/−^ mice, focused on the optic disc (A). TEM pictures of ON transversal sections from 12 month old *Wfs1^+/+^* and *Wfs1* ^−/−^ mice (B). RGC axonal densities counted in TEM pictures (C). Average values are presented +/− SEM. (n = 3 *Wfs1* ^+/+^, n = 4 *Wfs1* ^−/−^).

To evaluate the loss of RGC axonal fibers, eye fundi of 13 month mice were imaged with focus on the optic disc (Figure 4A). RPE aspect appeared similar in all mice, forming a uneven and blurry grey background behind the retinal tissue. Optic disc size and color were variable between individuals with occasional optic disc pallor as shown in Figure 4A. To establish if RGC axons were lost in mutant mice, transversal sections were analyzed by electron microscopy at the age of 12 months (Figure 4B). Axonal density was reduced by 13 percents in *Wfs1* mutants, although not significantly, could suggest a tendency for RGC axonal loss in the optic nerve (Figure 4C).

**Figure 5:**
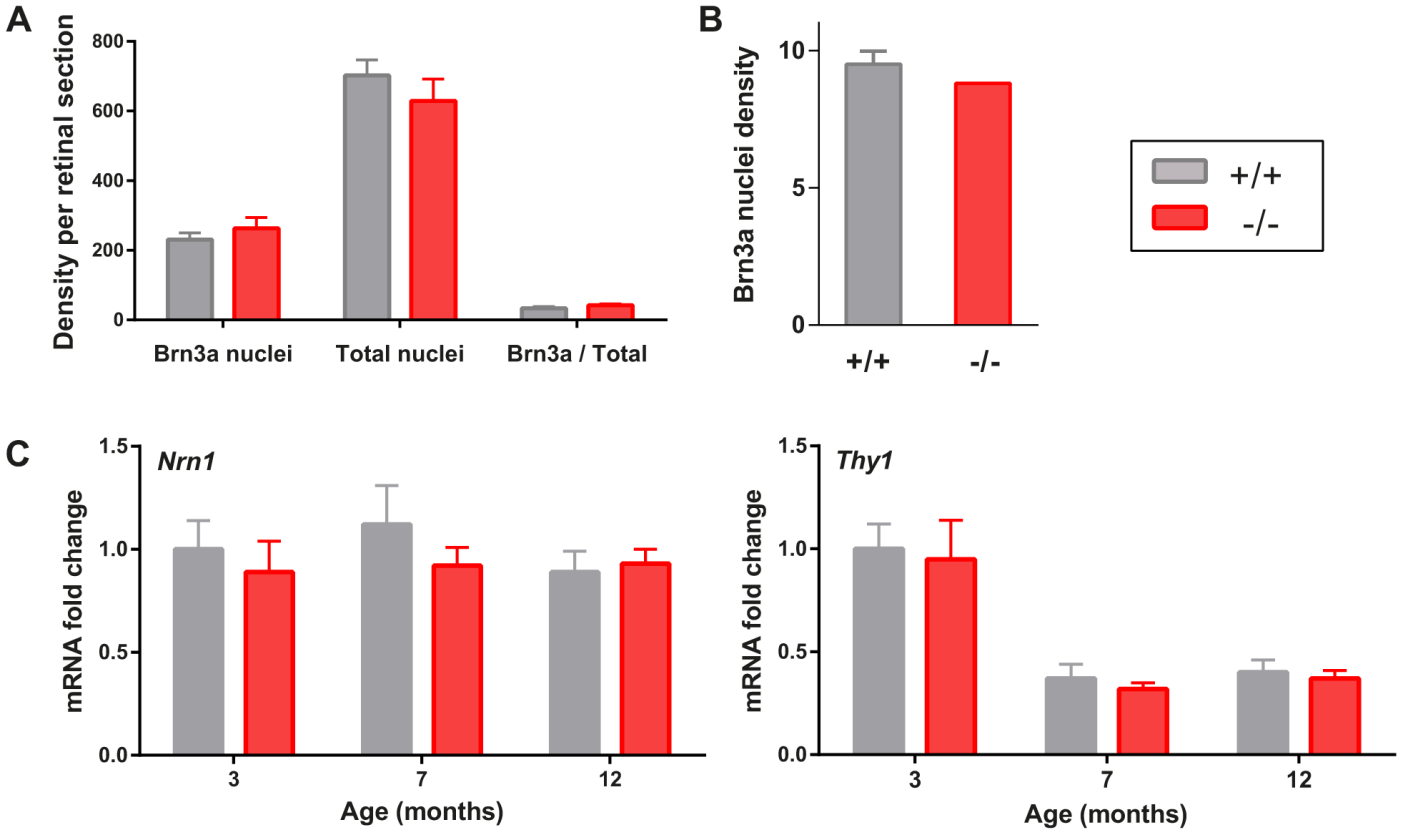
RGC cell density in *Wfs1* ^−/−^ mice. Brn3a and total nuclei were counted in nerve fiber layer of 12 month retinal sections (A). RGC cell density is given as average Brn3a cell nuclei in 1000 pixel segments of retina (B). *Thy1* and *Nrn1* relative gene expressions were quantified in retinal tissue at 3 (n = 4 *Wfs1* ^+/+^, n = 4 *Wfs1* ^−/−^), 7 (n = 5 *Wfs1* ^+/+^, n = 7 *Wfs1* ^−/−^) and 12 months (n = 8 *Wfs1* ^+/+^, n = 8 *Wfs1* ^−/−^) (C). Average values are presented +/− SEM, with statistical significance (*) indicated if p<0.05.

To determine if RGC were directly involved in VEP alterations, RGC were labeled by immunochemistry. No reduction in the number of nuclei and of Brn3a expressing RGC was found in sections including optic nerve papilla (Figure 5A). Density of Brn3a positive RGC along the retina was unchanged in normal and mutant mice, respectively with 8.8 and 9.5 cells/1000 pixels (p = 0.26 in Student bilateral mean comparison test) (Figure 5B). Moreover the retinal structure was preserved in mutant retina (Figure S1). To confirm histological observations, *Nrn1 a*nd *Thy1* mRNA levels were assessed by quantitative PCR in 3, 7 and 12 month old retinas (Figure 5C). *Nrn1* and *Thy1* are indeed among the most downregulated genes in mouse retinas after optic nerve transection and subsequent RGC loss [31]. *Nrn1* and *Thy1* retinal gene expressions were not significantly modified by *Wfs1* disruption at 3, 7 and 12 months. Thus RGC specific transcription was not reduced in *Wfs1* ^−/−^ mutant mouse, supporting the absence of significant RGC loss.

**Figure 6:**
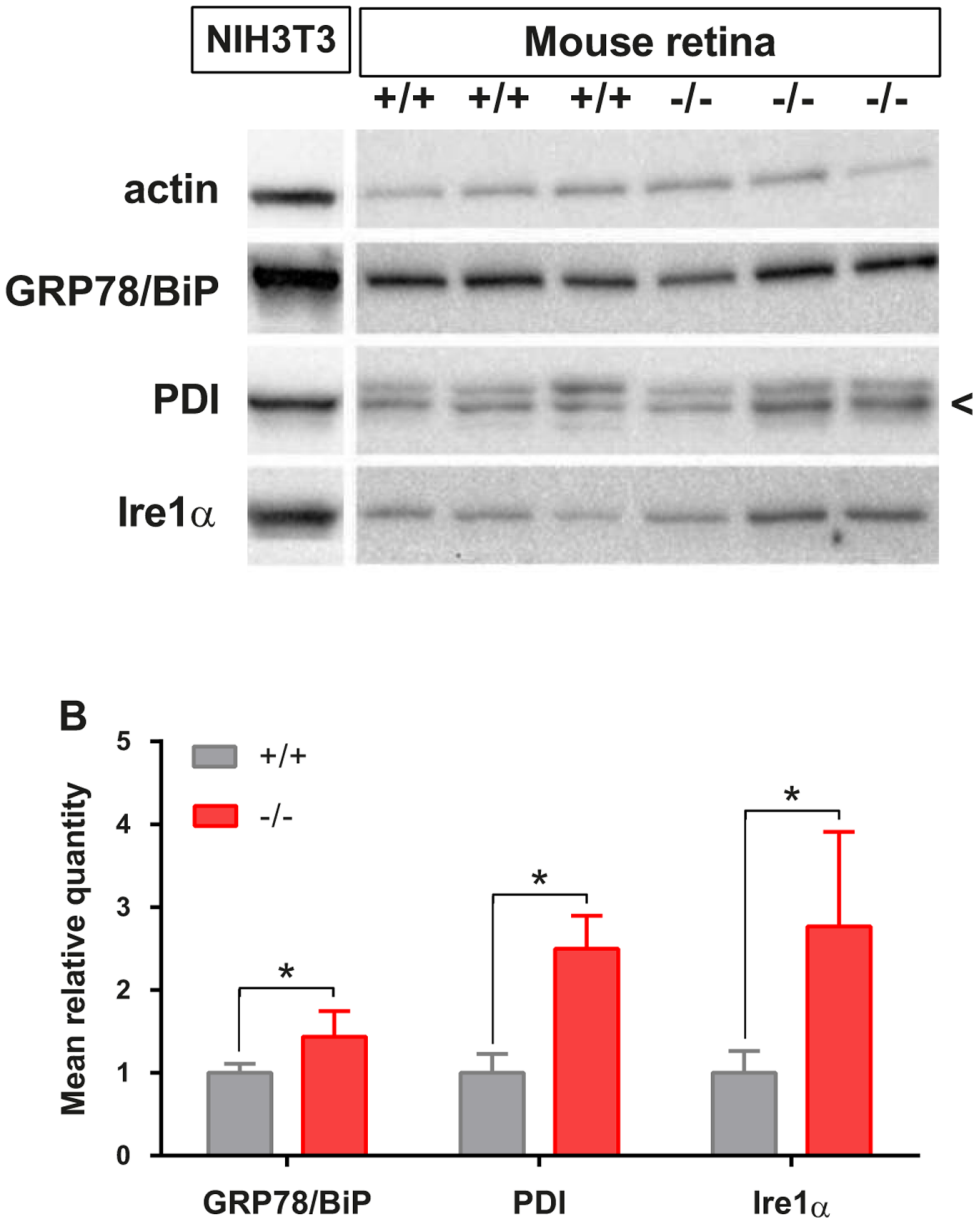
Retinal ER stress and UPR evaluation in *Wfs1^−/−^* mice. Immunoblots (A) detected GRP78/BiP, PDI, IRE1, and beta actin in protein lysates of 12 month old *Wfs1^+/+^* (n = 3) and *Wfs1^−/−^* (n = 3) mouse retinas and in mouse NIH3T3 fibroblasts treated with thapsigargin. Mean relative quantities for each protein according to *Wfs1* genotype were obtained after normalization with beta actin values. Significance (*) is indicated when p<0.05.

**Figure 7:**
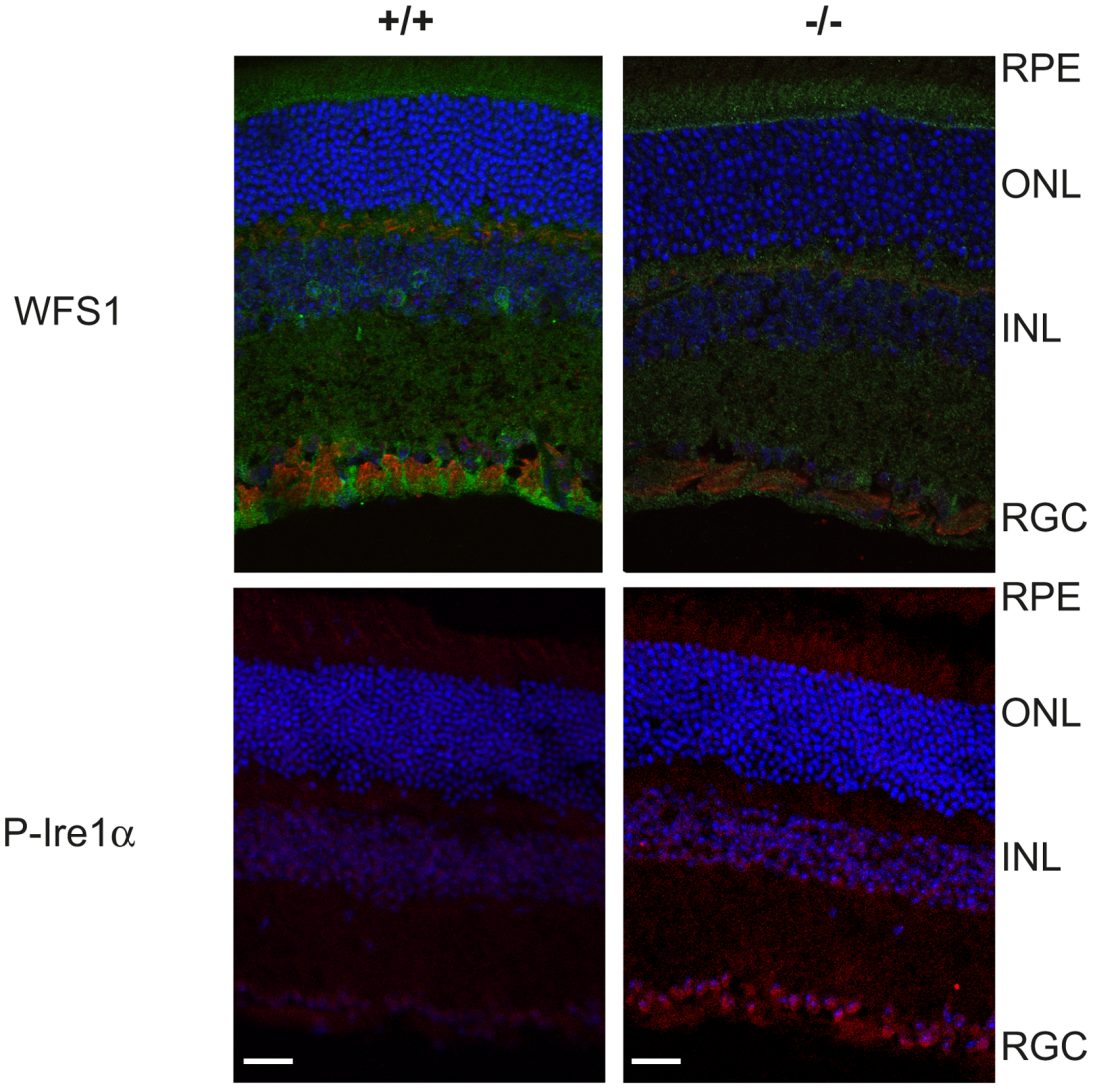
P-Ire1α activation correlates with WFS1 cell expression. Cryosections of retina from 12 month old *Wfs1^+/+^* and *Wfs1^−/−^* mouse were immunostained with anti-WFS1 antibody (green in top image) and NF200 (red in top image) or anti-P-Ire1α antibody (red in bottom image). DAPI was used for staining of cell nuclei (blue). RPE, retinal pigment epithelium; ONL, outer nuclear layer; INL, inner nuclear layer, RGC, retinal ganglion cells. Scale bars = 50 µm.

Since RGC population was preserved in *Wfs1^−/−^* mouse retina, we monitored the UPR signaling of the endoplasmic reticulum by western-blot (Figure 6). Thapsigargin-treated NIH3T3 mouse fibroblasts were used as a control of ER stress markers: GRP78/BiP, PDI and Ire1α. After normalization by beta actin protein levels, *Wfs1*-deficient retinas presented significantly increased relative levels of GRP78/BiP, Ire1 alpha and PDI proteins, in all animals tested. To investigate which cells in mutant retina express ER stress we performed immunostaining of phospo-Ire1 and WFS1 (Figure 7). Interestingly, WFS1 expressing cells as retinal ganglion cells and some interneurons seem to overexpress phospo-Ire1. Real time PCR analysis revealed that the mRNA level of GRP78 and spliced Xbp1 were slightly elevated in mutant retinas, but the effect did not reach statistical significance (p = 0,07 for sXbp1) (Figure S2). The mRNA levels of CCAAT-enhancer-binding protein homologous protein (CHOP) transcription factor can be considered as unchanged (Figure S2). *Wfs1* deficiency thus induced UPR signaling in mutant mouse retina, as an adaptative process that can protect retinal cells from cell death.

## Discussion

In order to describe a mouse model for Wolfram syndrome optic atrophy, we explored visual function of *Wfs1* deficient mice from 3 to 12 months of age. The longitudinal study of *Wfs1^−/−^* mutant mice depicted from the age of 9 months a reduction of VEP signaling associated to a functional alteration of inner retinal function, with preserved visual acuities. Further biochemical analysis at 12 months indicated a triggered UPR signaling in *Wfs1*-deficient retina, possibly as a protective mechanism of retinal and optic nerve damages.

Since retinal fundus examination at 12 months didn’t disclose systematically pale optic discs in *Wfs1^−/−^* animals, we analyzed axonal fibers by transmission electron microscopy at 12 months. Ultrastructural observation of ON transversal sections indicated a 13% loss of RGC axons that could partially support the reduction of VEP amplitude. No particular swelling, change of myelination, autophagic pattern was noted, confirming the global preservation of RGC axons in *Wfs1^−/−^* optic nerves. However the preservation of RGC density in *Wfs1*-deficient mouse retinas could imply functional changes in these cells. Structural and/or functional alterations in RGCs involving metabolic changes or impairment of axonal transport with full preservation of axons can be an explanation of the loss of VEP [32].

As inner retinal function was found impaired by ERG at the same ages, it could induce VEP alterations associated to healthy RGC. Retinal physiology of mutant *Wfs1* mice could differ from human WS patients given the difference in Wolframin expression pattern. Indeed, compared to a specific localization in human ganglion cell layer, Wolframin expression is predominantly expressed in mouse retinal pigment epithelium, inner retina and RGC [16]. Wolframin lack in *Wfs1^−/−^* inner retina may have led to significant changes in the propagation of light information in *Wfs1* disrupted mice, from bipolar cells to RGC and consequently from ERG b-wave to the VEP.

In addition, as central nervous system atrophy is found in WS patients [3], [33], [34], possible central degeneration in *Wfs1^−/−^* mice could contribute to the changes observed in VEP analysis, independently from retinal dysfunctions. *Wfs1* mRNA was shown to be enriched in excitatory neuronal cells of layer2/3 of the visual cortex in a subset of excitatory neurons responsive to selective visual stimuli [35]. Visual-derived brain signaling measured by VEP could therefore also be reduced by the absence of Wolframin in the visual cortex.

Optokinetic tracking reflex measured for visual acuity and contrast sensitivity was expected to be impaired in *Wfs1^−/−^* mutant mice as a consequence of retinal and visual system dysfunction. Nevertheless our longitudinal analysis of *Wfs1* mutant mice didn’t demonstrate any significant alteration of this reflex. Anatomically, this reflex is mediated by neurons from the retina, diencephalon and midbrain, pons and dorsal medulla [36]. From these observations, we can conclude that axonal damage to the optic nerve or visual pathways occurred, despite normal visual function which can be maintained due to the ability of the visual system to compensate. Compensation could occur based on changes of the primary visual cortex as it has been shown for balancing visual field defects [37]. Finally, the approach used in oculomotor phenotyping, by monitoring the mouse reflexive head at specific contrasts and frequencies, could be reinforced by evaluating subtil parameters such as chromatic discrimination, directional OKR with namely vertically moving stimulus and relatively small ocular movements followed by the positions of the pupil in oculography [38].

The moderate effects associated to *Wfs1* deficiency could also be due to *Wfs1* interaction with the genetic background [39]. Indeed, the pancreatic phenotype of *Wfs1^−/−^* mutant mouse is highly dependent upon genetic background, as the [(129SvxB6)xB6]F2 mutant mice present severe diabetes, while *Wfs1^−/−^* deficiency on the C57Bl/6J background induces only glucose intolerance [20]. Accordingly genetic inheritance from C57Bl/6J strain could somehow mask a more severe visual impairment in *Wfs1*-deficient mice. Furthermore, although WFS1 was not detected in the mutant mouse with mWfs1-Nter antibody, we cannot totally exclude the presence of a smaller truncated WFS1 protein with hypomorphic expression due to the lack of a specific mWfs1-Cter antibody.

To decipher if UPR signaling was activated in *Wfs1* mutant mice, we measured UPR markers BiP/GRP78, PDI and Ire1α in 12 month old *Wfs1* mutant retinas and found a significant increase of their protein expression. This result agrees with the UPR activation found in *Wfs1^−/−^* mouse pancreatic beta cells and with its absence in other tissues not affected by Wolfram syndrome, as shown in heart, skeletal muscle and adipose tissue [21], [27]. Importantly *Wfs1*-deficient retinas present in average higher expression level of Ire1α signaling pathway, suggesting a targeted activation of the UPR, but not of the other ER stress sensors, ATF6 and PERK (Figure S3) [40] [41]. We showed that autophagic marker LC3 seems to be increased in retinal ganglion cell in 12 month old *Wfs1* mutant retinas (Figure S4).

Paradoxically, autophagy can serve to protect cells [42], [43] but may also contribute to cell damage [44]. It was reported that the Ire1α signaling pathway directly activates c-Jun N-terminal kinase (JNK) [45], [46], an important inflammatory signaling mediator. Activated Ire1α and ER stress in various cells plays an important role in the pathogenesis of several diseases, including obesity, type 2 diabetes and cancer suggesting that ER stress-induced inflammation contributes substantially to disease progression. It is also interesting to note that the proapoptotic CHOP expression is not different in control and mutant retinas, suggesting that no significant apoptosis occur in *Wfs1^−/−^* retina. Thus in spite of VEP alteration, the protective function of UPR response, namely through Ire1α signaling could play pivotal roles in protecting RGC against the cell death in mutant retinas. This hypothesis is supported by previous work on models of neurodegenerative disease showing that ER stress inhibits neuronal death by promoting autophagy [47], [48].

In summary, the longitudinal study of *Wfs1*-deficient mouse disclosed a significant dysfunction of the visual system, associated with a protective ER stress in the retina, but without major loss of RGC. However this model is not a great model to investigate optic atrophy in Wolfram syndrome. An alternative strategy to study the mechanisms of visual impairment in Wolfram syndrome is iPS cells. IPS cells enable the creation of “human disease models” since they can be derived from affected patients. The iPS cells from the WS patients will serve as a true *in vitro* model reflecting the same pathological features as the one encountered *in vivo*. IPS cells from patients differentiated in retinal ganglion cells will provide a patient specific model to study the pathophysiology and discover drugs to treat the optic atrophy in Wolfram disease.

## Supporting Information

Figure S1 Histologic analysis of wild-type and mutant retina. The cryosections of 12 month old wild-type retina (left) and *Wfs1^−/−^* retina (right) were stained with haematoxylin and eosin. RPE, retinal pigment epithelium; ONL, outer nuclear layer; INL, inner nuclear layer; RGC, retinal ganglion cells. Scale bars = 100 µm.
(TIF)

Figure S2 Real time PCR of CHOP, GRP78 and Spliced Xbp1. Bar graph of real time PCR of genes CHOP, GRP78 and spliced Xbp1(sXbp1) showing the mean +/− SEM in delta CT values (normalized against L27, (left)) and fold change (right) in 12 month old *Wfs1^+/+^* (n = 4) and *Wfs1^−/−^* (n = 6) mice retinas. Fold change in expression was calculated using the double delta Ct method assuming 100% efficiency.
(TIF)

Figure S3 Retinal ER stress evaluation. Western blotting using anti-β-actin, anti-PERK, anti-phospho-PERK (P-PERK) and anti-ATF6 in protein lysates of 12 month old *Wfs1^+/+^* (n = 3) and *Wfs1^−/−^* (n = 3) mouse retinas and in mouse NIH3T3 fibroblasts treated with thapsigargin.
(TIF)

Figure S4 Expression of LC3 in mouse retina. Cryosections of retina from 12 month old *Wfs1^+/+^* and *Wfs1^−/−^* mouse were immunostained with anti-LC3 (red). DAPI was used for staining of cell nuclei (blue) (A). Quantification of fluorescence intensity of LC3 signal (red) in RGC layer in +/+ and −/− retinas. The range of fluorescence intensity per pixel in the RGC layer was from 0–255∶0 =  black, 255 =  saturated. Data are means +/− SEM (B). Western blot analysis showing levels of LC3-I and LC3-II and actin in retinas from wild type (+/+) and mutant mice (−/−). Right panel, quantification of protein in signal intensities showing LC3-II protein levels in extracts from +/+ and −/− retinas. Values denote means +/−SEM (n = 2) (C). RPE, retinal pigment epithelium; ONL, outer nuclear layer; INL, inner nuclear layer, RGC, retinal ganglion cells. Scale bars = 50 µm.
(TIF)
