# Supplementary figures and images for: Impairment of Visual Function and Retinal ER Stress Activation in Wfs1-Deficient Mice
Source: PLoS One. 2014 May 13;9(5):e97222. doi: 10.1371/journal.pone.0097222 (PMC4019519; doi:10.1371/journal.pone.0097222)

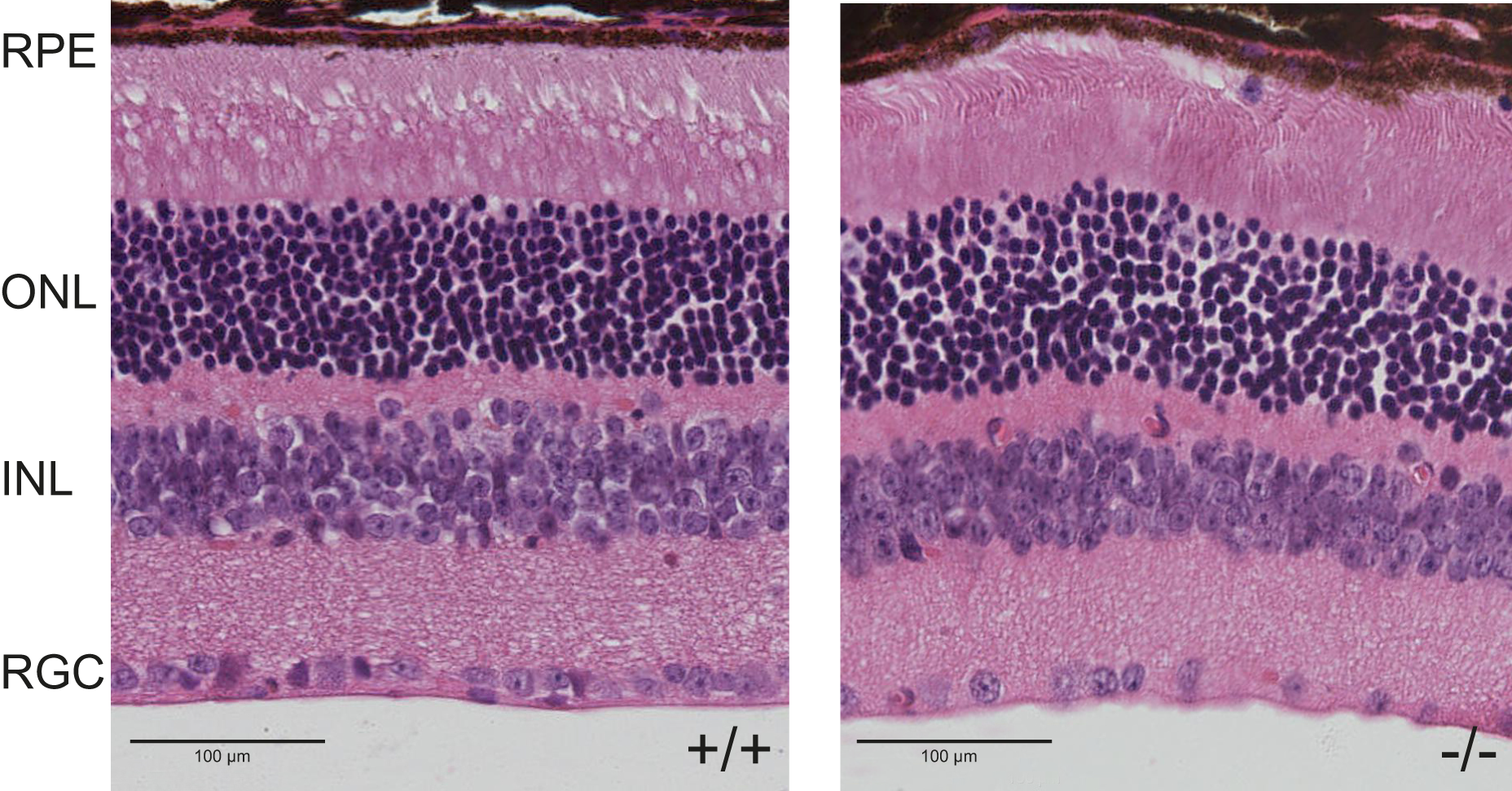

Supplement: Figure S1 — Histologic analysis of wild-type and mutant retina. The cryosections of 12 month old wild-type retina (left) and Wfs1−/− retina (right) were stained with haematoxylin and eosin. RPE, retinal pigment epithelium; ONL, outer nuclear layer; INL, inner nuclear layer; RGC, retinal ganglion cells. Scale bars = 100 µm. (TIF) [file pone.0097222.s001.tif]

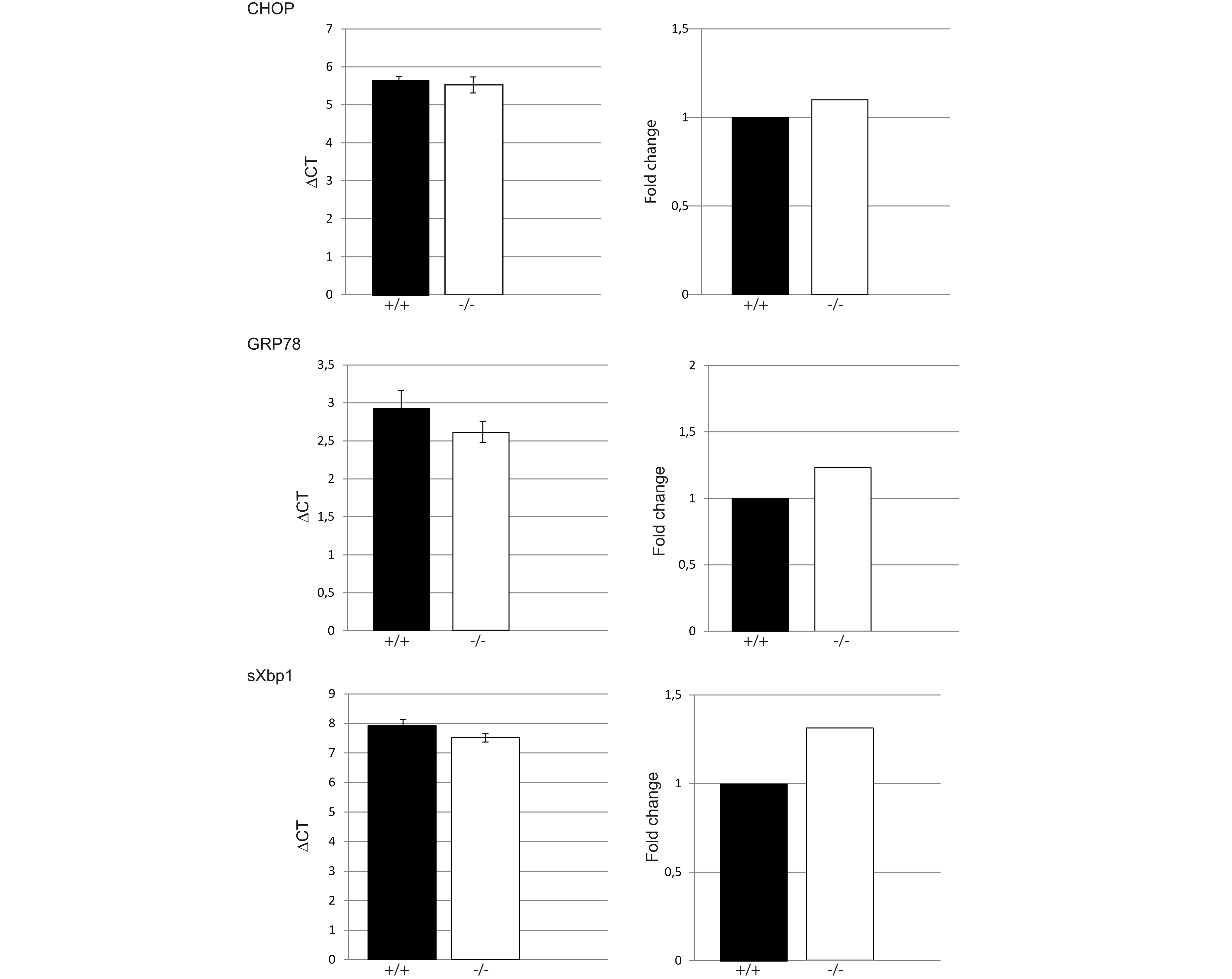

Supplement: Figure S2 — Real time PCR of CHOP, GRP78 and Spliced Xbp1. Bar graph of real time PCR of genes CHOP, GRP78 and spliced Xbp1(sXbp1) showing the mean +/− SEM in delta CT values (normalized against L27, (left)) and fold change (right) in 12 month old Wfs1+/+ (n = 4) and Wfs1−/− (n = 6) mice retinas. Fold change in expression was calculated using the double delta Ct method assuming 100% efficiency. (TIF) [file pone.0097222.s002.tif]

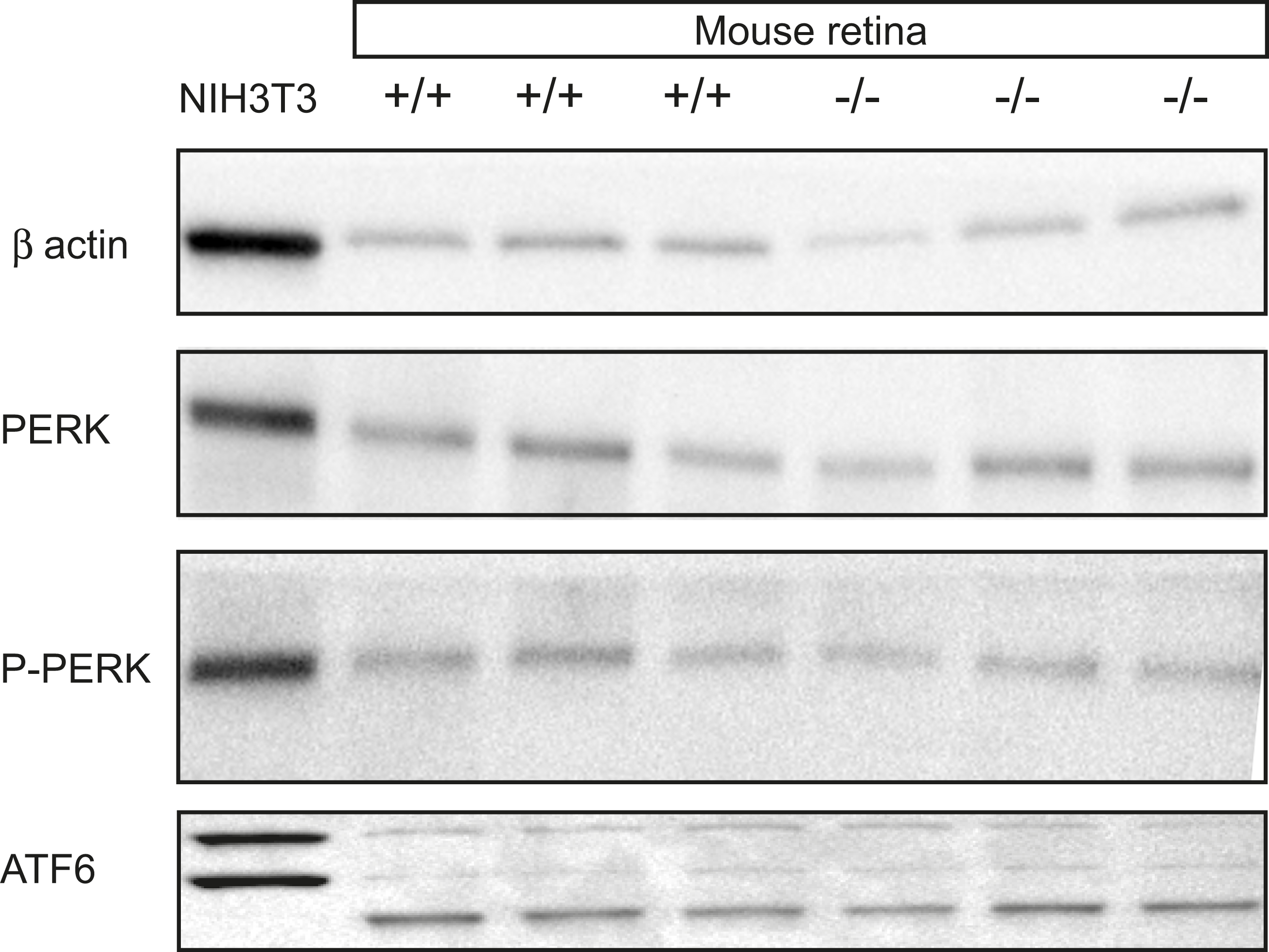

Supplement: Figure S3 — Retinal ER stress evaluation. Western blotting using anti-β-actin, anti-PERK, anti-phospho-PERK (P-PERK) and anti-ATF6 in protein lysates of 12 month old Wfs1+/+ (n = 3) and Wfs1−/− (n = 3) mouse retinas and in mouse NIH3T3 fibroblasts treated with thapsigargin. (TIF) [file pone.0097222.s003.tif]

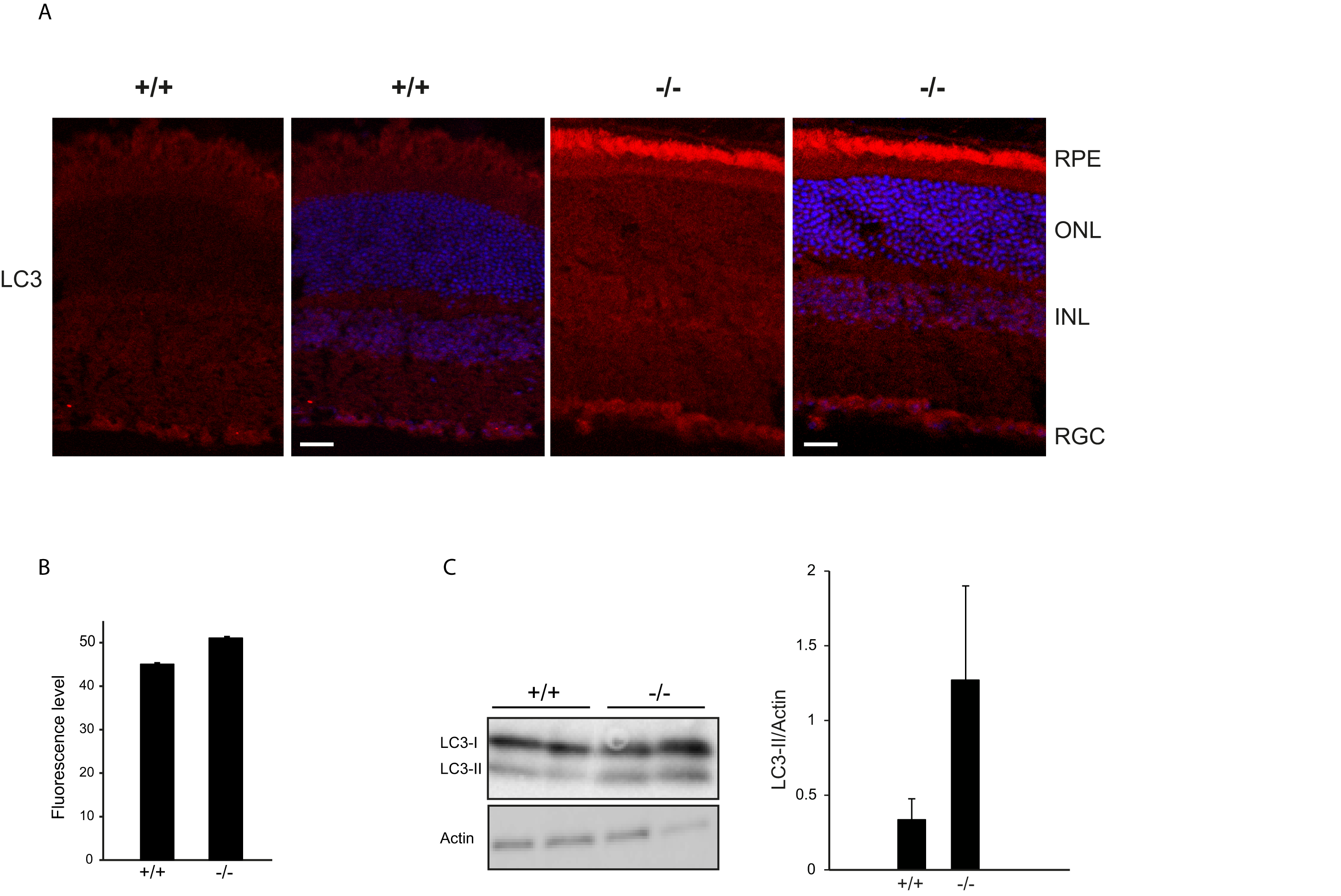

Supplement: Figure S4 — Expression of LC3 in mouse retina. Cryosections of retina from 12 month old Wfs1+/+ and Wfs1−/− mouse were immunostained with anti-LC3 (red). DAPI was used for staining of cell nuclei (blue) (A). Quantification of fluorescence intensity of LC3 signal (red) in RGC layer in +/+ and −/− retinas. The range of fluorescence intensity per pixel in the RGC layer was from 0–255∶0 = black, 255 = saturated. Data are means +/− SEM (B). Western blot analysis showing levels of LC3-I and LC3-II and actin in retinas from wild type (+/+) and mutant mice (−/−). Right panel, quantification of protein in signal intensities showing LC3-II protein levels in extracts from +/+ and −/− retinas. Values denote means +/−SEM (n = 2) (C). RPE, retinal pigment epithelium; ONL, outer nuclear layer; INL, inner nuclear layer, RGC, retinal ganglion cells. Scale bars = 50 µm. (TIF) [file pone.0097222.s004.tif]
